# Evaluation of sodium butyrate and nutrient concentration for broiler chickens

**DOI:** 10.1016/j.psj.2021.101456

**Published:** 2021-08-30

**Authors:** J.J. Mallo, C. Sol, M. Puyalto, C. Bortoluzzi, T.J. Applegate, M.J. Villamide

**Affiliations:** ⁎ Norel S.A., Madrid 28007, Spain; † Polytechnic University of Madrid, Madrid, Spain; ‡ Department of Poultry Science, University of Georgia, Athens, GA 30602, USA

**Keywords:** broilers, sodium butyrate, digestibility, intestinal microbiota, volatile fatty acids

## Abstract

The relation between nutrition and intestinal health is a subject with an increasing interest in research, as nutritionists need knowledge about how formulation affects different parameters in the gastrointestinal tract (**GIT**). That is why 4 trials were conducted to evaluate the effect of nutrient concentration and a feed additive (sodium butyrate protected with sodium salts of palm fatty acid distillates (PSB, Gustor N'RGY produced by Norel S.A., Spain, dosed at 1 kg/t), on performance, diet digestibility, intestinal morphology, volatile fatty acid concentration (**VFA**) in the GIT and intestinal microbiota of broiler chickens, when fed diets with different energy and amino acids concentration. Control diets, C, with the recommended metabolizable energy (**ME**) and ideal amino acid (**AA**) composition; Reduction 1, R1, C – 60 kcal ME and – 2.3% AA and Reduction 2, R2, C – 120 kcal ME and – 4.6% AA) based on different feed ingredients (Corn Soy [**CS**] and Wheat Barley Soy (**WBS**) were formulated. All trials lasted 42 d. In trials 2 and 4, the nutrient dilution decreased performance of the animals. In all trials, PSB improved animal performance (growth or FCR), despite the different situations. In trials 1 and 4, animals receiving R1 diets and PSB showed similar performance to those receiving C diets without PSB. PSB improved Gross Energy metabolizability (69.94 vs. 72.55; *P*: 0.02). Nutrient concentration affected histology results in T2 (ileum) and T3 (jejunum); PSB showed effects in T2 (jejunum, ileum) and in T3 (jejunum). In T1, PSB affected VFA in duodenum, jejunum, and ileum, changing the profile depending on diet nutrient concentration. PSB altered microbiology in caecum of animals in T2. It can be concluded that the dilution of ME and AA concentration of the diet impairs animal performance, influences intestinal microbiota and affects intestinal histology. PSB improves animal performance, increases gross energy metabolizability, steers intestinal microbiota and alters VFA concentrations in the intestine. The addition of PSB may help the animal to counteract the negative effects of diluted diets.

## INTRODUCTION

One of the most active fields in poultry research is the evaluation of alternatives to antibiotics in feeds. Ever since antibiotics were banned as growth promoters in the EU (Castanon, 2007), there is a global trend to produce animal protein using as little chemotherapeutics as possible (Gadde et al., 2017). The poultry nutritionist is nowadays challenged to produce at the most efficient cost, and it is necessary to consider not only ingredient cost, but also how this situation may affect the gastrointestinal health of animals (Yegani and Korver, 2008).

There are many feed additives and active feed ingredients that have several positive effects on the gastrointestinal tract (**GIT**) ambience of the animal: enzymes, essential oils, short and medium chain fatty acids and their salts, pre- and probiotics, etc. (Gadde et al., 2017). However, the mechanism of action is not very well-described in many cases, and results are not always consistent. Besides, the research done with them is often narrowed to a specific situation, making it difficult for the professional to evaluate whether the observed effects could be replicated in field conditions, with different genetic lines, feed ingredients, formulations, etc.

Butyric acid is a short chain fatty acid with different positive effects on the animal. Its derivatives (sodium and calcium salts, as well as mono, di- and triglycerides) are commonly used in animal nutrition. Among them, the best described is sodium butyrate, whose effects in the animals vary from producing longer GIT villi (Chamba et al., 2014) to modifying bacterial populations in the intestine and caecum (Fernández-Rubio et al., 2009; Bortoluzzi et al., 2017). It is not clear, though, if all the effects described with sodium butyrate can also be seen with other butyrate derivatives (Yang et al., 2018).

Thus, in order to gain further insight into the consistency of the results of a nonmedicated active feed ingredient, by evaluating if it can compensate eventual nutritional deficiencies of the diet, and if so, how and to what extent, four trials were conducted to test the effect of nutrient concentration and an active feed ingredient addition (sodium butyrate protected with sodium salts of palm fatty acid distillates (PSB, Gustor N'RGY produced by Norel S.A., Madrid, Spain, dosed at 1 kg/t), on performance, protein and energy utilization, intestinal morphology, intestinal volatile fatty acid concentration (**VFA**) in the gastrointestinal tract and intestinal microbiota of animals. In these trials, the animals were fed diets that differed in nutrient concentrations and feed ingredients: Trials 1, 2, and 3 were conducted under standard European Union production practices, and trial 4 was run under standard North American production practices; besides animals in trials 1 and 3 received a wheat, barley, and soy based diet, whereas animals in trials 2 and 4 received a corn and soy based diet. At the same time, in all trials, the nonmedicated active feed ingredient was evaluated with a factorial design (application-not application). The hypothesis of these studies was that the supplementation of sodium butyrate to nutritionally reduced diets would counteract the effect on performance of broiler chickens by improving the energy and nutrient utilization and modulating the intestinal health.

## MATERIALS AND METHODS

### Housing, Birds, and Treatments

Experiments were performed at the Experimental Unit of Norel in León, University of Lleida and Purdue University, and received prior approval from the Animal and Human Experimental Ethical Committee Authorities responsible for every Institution. A summary of the setup of the trials can be found in Table 1.

Diets for the 4 trials, supplied ad-libitum as mash, were formulated with different nutrient concentration as follow: Control diets (**C**), had the recommended metabolizable energy (**ME**) and ideal amino acid (**AA**) composition according to Fedna and Mateos (2008) (trials 1–3) or NRC (1994) (trial 4). Two other diets were formulated decreasing their nutrient densities: reduction 1 (R1), had 60 kcal less ME and 2.3% less AA than C, and reduction 2 (R2), which had 120 kcal less ME and 4.6% less AA than C. Additionally, the diets differed in feed ingredients among trials: corn and soy (**CS**) were used as main ingredients in trial 2 (Table 3) and 4 (Table 5), and wheat, barley, and soy (**WBS**) in trial 1 (Table 2) and 3 (Table 4). Nutrient densities were modified adjusting the levels of the feed ingredients used in the formulation and reducing essential amino acids such as L-lysine, DL- methionine, and L-threonine.

**Table 1:** Summary of the experimental designs of the four trials.

Trial	Trial 1		Trial 2		Trial 3		Trial 4	
Design: Nutrient concentration × PSB addition	2 × 2		2 × 2		3 × 2		3 × 2	
n° replicates (animals per replicate)/treatment	5 replicates (10 animals)/treatment		5 replicates (10 animals)/treatment		6 replicates (7 animals)/treatment		8 replicates (46 animals)/treatment	
Main ingredients	A) Wheat + Barley + Soy		B) Corn + Soy		A) Wheat + Barley + Soy		B) Corn + Soy	
Treatments	Nutrient	Additive	Nutrient	Additive	Nutrient	Additive	Nutrient	Additive
T1	Standard	NO	Standard	NO	Standard	NO	Standard	NO
T2	Reduction 1	NO	Reduction 1	NO	Reduction 1	NO	Reduction 1	NO
T3	-	-	-	-	Reduction 2	NO	Reduction 2	NO
T4	Standard	PSB	Standard	PSB	Standard	PSB	Standard	PSB
T5	Reduction 1	PSB	Reduction 1	PSB	Reduction 1	PSB	Reduction 1	PSB
T6	-	-	-	-	Reduction 2	PSB	Reduction 2	PSB

PSB: protected sodium butyrate 1 kg/t.

Nutrient concentration:

- Standard: 3,000 kcal AMEn/kg; 22% CP; 11.6% Lys;

- Reduction 1: Standard – 60 kcal/kg; −2.3% aa;

- Reduction 2: Standard – 120 kcal/kg; −4.6% aa.

**Table 2:** Composition of the experimental diets of trial 1.

	1–21 d		21–42 d	
Ingredient, %	Control	Reduced 1	Control	Reduced 1
Wheat	49.16	49.17	54.64	58.06
Barley	5.00	7.50	5.00	7.50
Soybean meal, 48% CP	36.45	35.02	30.96	26.34
Soybean oil	5.71	4.69		
Animal fat			6.26	4.80
Dicalcium phosphate	1.70	1.69	1.45	1.47
Calcium carbonate	0.77	0.78	0.63	0.64
Sodium chloride	0.40	0.40	0.35	0.35
L-lysine HCl	0.15	0.11	0.07	0.15
DL-methionine	0.26	0.24	0.18	0.18
L-threonine	0.04	0.03	0.04	0.07
Coccidiostat	0.05	0.05	0.05	0.05
Choline chloride	0.03	0.03	0.05	0.05
Vitamin premix- broilers^1^	0.30	0.30	0.30	0.30
Formulated nutrient content				
ME Kcal/Kg	3,000.00	2,940.00	3,050.00	2,990.00
CP, %	22.02	21.98	20.00	18.92
Lysine, %	1.27	1.24	1.08	1.05
Thr, %	0.83	0.82	0.76	0.74
Met+Cys, %	0.93	0.92	0.81	0.78
aP, %	0.45	0.45	0.40	0.40
Ca, %	0.93	0.93	0.80	0.80
Na, %	0.17	0.17	0.15	0.15

1 Supplied the following per kilogram of diet: supplied per kg of diet: vitamin A, 12,500 IU; vitamin D_3_, 2,500 IU; vitamin K_3_, 2.65 mg; vitamin B_1_, 2 mg; vitamin B_2_, 6 mg; vitamin B_12_, 25 µg; vitamin E, 30 IU; biotin, 0.0325 mg; folic acid, 1.35 mg; pantothenic acid 12 mg; niacin, 50 mg; iron from ferrous sulfate, 50.1 mg; copper from copper sulfate, 8 mg; manganese from manganese oxide, 100 mg; zinc from zinc oxide, 75 mg; iodine from ethylene diamine dihydroidide, 0.35 mg; selenium from sodium selenite, 0.15 mg.

**Table 3:** Composition of the experimental diets of trial 2.

	1–21 d		21–42 d	
Ingredient, %	Control	Reduced 1	Control	Reduced 1
Corn	52.11	51.12	58.50	57.64
Soybean meal, 48% CP	38.71	37.92	33.05	32.32
Soybean oil	4.83	4.80	4.70	4.70
Dicalcium phosphate	1.90	1.90	1.31	1.31
Limestone	1.30	1.30	1.40	1.40
Sodium chloride	0.35	0.35	0.35	0.35
L-lysine HCl	0.10	0.09	0.08	0.07
DL-methionine	0.18	0.17	0.08	0.07
L-threonine	0.04	0.03	0.02	0.01
Coccidiostat	0.05	0.05	0.05	0.05
Choline chloride	0.03	0.03	0.03	0.03
Vitamin premix- broilers^1^	0.30	0.30	0.30	0.30
Sepiolite	0.10	1.94	0.10	1.85
Formulated nutrient content				
ME Kcal/Kg	3,000.00	2,940.00	3,050.00	2,990.00
CP, %	21.00	20.78	20.00	19.27
Lysine, %	1.27	1.24	1.10	1.07
Thr, %	0.85	0.83	0.76	0.74
Met+Cys, %	0.86	0.84	0.78	0.76
aP, %	0.45	0.45	0.40	0.40
Ca, %	0.93	0.93	0.80	0.80
Na, %	0.17	0.17	0.15	0.15

1 Supplied the following per kilogram of diet: supplied per kg of diet: vitamin A, 12,500 IU; vitamin D_3_, 2,500 IU; vitamin K_3_, 2.65 mg; vitamin B_1_, 2 mg; vitamin B_2_, 6 mg; vitamin B_12_, 25 µg; vitamin E, 30 IU; biotin, 0.0325 mg; folic acid, 1.35 mg; pantothenic acid 12 mg; niacin, 50 mg; iron from ferrous sulfate, 50.1 mg; copper from copper sulfate, 8 mg; manganese from manganese oxide, 100 mg; zinc from zinc oxide, 75 mg; iodine from ethylene diamine dihydroidide, 0.35 mg; selenium from sodium selenite, 0.15 mg.

**Table 4:** Composition of the experimental diets of trial 3.

	1–21 d			21–42 d		
Ingredient, %	Control	Reduced 1	Reduced 2	Control	Reduced 1	Reduced 2
Wheat	49.16	49.17	47.84	54.64	58.06	56.82
Barley	5.00	7.50	10.00	5.00	7.50	10.00
Soybean meal, 48% CP	36.45	35.02	34.81	30.96	26.34	26.12
Soybean oil	5.71	4.69	3.78			
Animal fat				6.26	4.80	3.82
Dicalcium phosphate	1.70	1.69	1.68	1.45	1.47	1.46
Calcium carbonate	0.77	0.78	0.78	0.63	0.64	0.65
Sodium chloride	0.40	0.40	0.40	0.35	0.35	0.35
L-lysine HCl	0.15	0.11	0.09	0.07	0.15	0.12
DL-methionine	0.26	0.24	0.23	0.18	0.18	0.17
L-threonine	0.04	0.03	0.01	0.04	0.07	0.06
Coccidiostat	0.05	0.05	0.05	0.05	0.05	0.05
Choline chloride	0.03	0.03	0.03	0.05	0.05	0.05
Vitamin premix- broilers^1^	0.30	0.30	0.30	0.30	0.30	0.30
Formulated nutrient content						
ME Kcal/Kg	3,000.00	2,940.00	2,880.00	3,050.00	2,990.00	2,930.00
CP, %	22.02	21.98	21.94	20.00	18.92	18.88
Lysine, %	1.27	1.24	1.22	1.08	1.05	1.03
Thr, %	0.83	0.82	0.80	0.76	0.74	0.72
Met+Cys, %	0.93	0.92	0.91	0.81	0.78	0.78
aP, %	0.45	0.45	0.45	0.40	0.40	0.40
Ca, %	0.93	0.93	0.93	0.80	0.80	0.80
Na, %	0.17	0.17	0.17	0.15	0.15	0.15

1 Supplied the following per kilogram of diet: supplied per kg of diet: vitamin A, 12,500 IU; vitamin D_3_, 2,500 IU; vitamin K_3_, 2.65 mg; vitamin B_1_, 2 mg; vitamin B_2_, 6 mg; vitamin B_12_, 25 µg; vitamin E, 30 IU; biotin, 0.0325 mg; folic acid, 1.35 mg; pantothenic acid 12 mg; niacin, 50 mg; iron from ferrous sulfate, 50.1 mg; copper from copper sulfate, 8 mg; manganese from manganese oxide, 100 mg; zinc from zinc oxide, 75 mg; iodine from ethylene diamine dihydroidide, 0.35 mg; selenium from sodium selenite, 0.15 mg.

**Table 5:** Composition of the experimental diets of trial 4.

	1–14 d			14–28 d			28–42 d		
Ingredient, %	Control	Reduced 1	Reduced 2	Control	Reduced 1	Reduced 2	Control	Reduced 1	Reduced 2
Corn	57.12	55.96	54.81	58.97	57.65	56.09	65.55	64.18	62.88
Soybean meal, 47.5% CP	35.70	34.87	34.11	33.29	32.74	32.21	27.16	26.74	26.11
Soybean oil	2.30	2.30	2.30	3.61	3.60	3.67	3.34	3.30	3.30
Monocalcium phosphate	1.80	1.81	1.82	1.25	1.26	1.27	1.16	1.17	1.18
Limestone	1.36	1.37	1.37	1.50	1.50	1.50	1.45	1.45	1.45
Sodium chloride	0.45	0.45	0.45	0.40	0.40	0.40	0.40	0.40	0.40
L-lysine HCl	0.32	0.31	0.30	0.17	0.16	0.15	0.18	0.17	0.16
DL-methionine	0.40	0.39	0.38	0.31	0.30	0.29	0.27	0.26	0.26
L-threonine	0.09	0.08	0.08	0.02	0.2	0.01	0.02	0.01	0.01
Vitamin premix- broilers^1^	0.35	0.35	0.35	0.35	0.35	0.35	0.35	0.35	0.35
Sulkafloc	0.10	2.08	4.00	0.1	2.00	4.03	0.1	1.94	3.87
Formulated nutrient content									
ME Kcal/Kg	3,050.00	2,990.00	2,930.00	3,150.00	3,090.00	3,030.00	3,200	3,140	3,080
CP, %	22.17	21.67	21.20	20.78	20.40	20.00	18.50	18.17	17.76
Lysine, %	1.43	1.39	1.36	1.24	1.21	1.18	1.09	1.06	1.04
Thr, %	0.94	0.91	0.89	0.83	0.81	0.79	0.74	0.72	0.70
Met+Cys, %	1.07	1.04	1.02	0.95	0.92	0.90	0.86	0.84	0.82
aP, %	0.50	0.50	0.5	0.38	0.38	0.38	0.35	0.35	0.35
Ca, %	0.95	0.95	0.95	0.90	0.90	0.90	0.84	0.85	0.85
Na, %	0.20	0.20	0.20	0.18	0.18	0.18	0.18	0.18	0.18

1 Supplied the following per kilogram of diet: supplied per kg of diet: vitamin A, 13,233 IU; vitamin D3, 6,636 IU; vitamin E, 44.1 IU; vitamin K, 4.5 mg; thiamine, 2.21 mg; riboflavin, 6.6 mg; pantothenic acid, 24.3 mg; niacin, 88.2 mg; pyridoxine, 3.31 mg; folic acid, 1.10 mg; biotin, 0.33 mg; vitamin B12, 24.8 µg; choline, 669.8 mg; iron from ferrous sulfate, 50.1 mg; copper from copper sulfate, 7.7 mg; manganese from manganese oxide, 125.1 mg; zinc from zinc oxide, 125.1 mg; iodine from ethylene diamine dihydroidide, 2.10 mg; selenium from sodium selenite, 0.25 mg.

**Table 6:** Effect of nutrient density reduction and protected sodium butyrate (PSB) inclusion on body weight and feed conversion ratio (FCR) of growing chickens at d 21 (Trials 1, 2, and 3) and at d 28 (Trial 4).

Nutrient	Additive	Body weight, Kg				FCR, Kg/Kg			
		Trial 1 d 21	Trial 2 d 21	Trial 3 d 21	Trial 4 d 28	Trial 1 d 21	Trial 2 d 21	Trial 3 d 21	Trial 4 d 28
		W-B-S	C-S	W-B-S	C-S	W-B-S	C-S	W-B-S	C-S
C	NO	0.77	0.73	0.83	1.37^A^	1.32	1.35	1.52	1.44
R 1	NO	0.76	0.75	0.82	1.31^BC^	1.28	1.39	1.54	1.44
R 2	NO	-	-	0.78	1.26^D^	-	-	1.61	1.47
C	PSB	0.81	0.80	0.82	1.35^AB^	1.26	1.36	1.50	1.42
R 1	PSB	0.79	0.77	0.89	1.35^AB^	1.27	1.39	1.50	1.44
R 2	PSB	-	-	0.82	1.30^CD^	-	-	1.53	1.49
SEM		0.012	0.020	0.024	0.015	0.018	0.021	0.107	0.013
*P*-value (Nutrient × Additive)		NS	NS	NS	0.004	NS	NS	NS	NS
Main effect mean									
Nutrient density	C	0.79	0.76	0.83^ab^	1.36^A^	1.28	1.35	1.51^B^	1.43^B^
	R 1	0.78	0.76	0.86^a^	1.33^B^	1.29	1.39	1.52^B^	1.44^B^
	R 2	-	-	0.80^b^	1.28^C^	-	-	1.57^A^	1.48^A^
SEM		0.008	0.014	0.017	0.011	0.013	0.015	0.075	0.009
*P*-value (Nutrient density)		NS	NS	0.0382	<0.001	NS	0.0972	0.0070	<0.001
PSB addition	NO	0.77	0.74	0.81	1.31	1.30	1.37	1.56	1.45
	PSB	0.80	0.78	0.85	1.33	1.27	1.38	1.51	1.45
SEM		0.008	0.014	0.014	0.039	0.013	0.015	0.061	0.045
*P*-value (PSB addition)		0.021	0.039	0.030	0.040	0.091	NS	0.003	NS

PSB: Protected sodium butyrate at 1 kg/t of feed.

C: Control diet: 3,000 kcal AMEn/kg; 22% CP; 11.6% Lys.

R1: Reduction 1 diet: control diet – 60 kcal/kg; −2.3% aa.

R2: Reduction 2 diet: control diet – 120 kcal/kg; −4.6% aa.

W-B-S: Wheat, barley and soy based diet.

C-S: Corn and soy based diet.

a–b–c Means with different superscripts differ significantly (*P* ≤ 0.05).

A–B–C Means with different superscripts differ significantly (*P* ≤ 0.01).

**Table 7:** Effect of nutrient density reduction and protected sodium butyrate (PSB) inclusion on final body weight (kg) and feed conversion ratio (FCR) of growing chickens fed for 42 d, in four different trials.

Nutrient	Additive	Final body weight, Kg				FCR, Kg/Kg			
		Trial 1	Trial 2	Trial 3	Trial 4	Trial 1	Trial 2	Trial 3	Trial 4
		W-B-S	C-S	W-B-S	C-S	W-B-S	C-S	W-B-S	C-S
Control	NO	2.29^ab^	2.04	1.94	2.81^a^	1.57	1.56	1.89	1.61
R 1	NO	2.19^b^	2.07	1.95	2.68^b^	1.58	1.61	1.95	1.63
R 2	NO	-	-	1.80	2.57^c^	-	-	2.13	1.69
C	PSB	2.21^b^	2.24	1.95	2.76^a^	1.54	1.55	2.01	1.60
R 1	PSB	2.34^a^	2.18	1.99	2.75^a^	1.54	1.58	1.82	1.63
R 2	PSB	-	-	1.82	2.67^b^	-	-	2.03	1.70
SEM		0.043	0.043	0.119	0.038	0.020	0.016	0.125	0.021
*P*-value (Nutrient × Additive)		0.0240	NS	NS	0.0030	NS	NS	NS	NS
Main effect mean									
Nutrient density	C	2.25	2.14	1.95	2.78^a^	1.56	1.56	1.95	1.60^c^
	R 1	2.27	2.13	1.97	2.71^b^	1.56	1.60	1.88	1.63^b^
	R 2	-	-	1.81	2.62^c^	-	-	2.08	1.69^a^
SEM		0.031	0.031	0.084	0.027	0.014	0.012	0.088	0.015
*P*-value (Nutrient density)		NS	NS	NS	<0.001	NS	0.030	NS	<0.001
PSB addition	NO	2.25	2.06	1.90	2.68	1.58	1.58	1.99	1.65
	PSB	2.28	2.21	1.92	2.73	1.55	1.57	1.96	1.64
SEM		0.031	0.031	0.068	0.082	0.014	0.012	0.072	0.051
*P*-value (PSB addition)		NS	0.004	NS	0.030	0.130	NS	NS	NS

PSB: Protected sodium butyrate at 1 kg/t of feed.

C: Control diet: 3,000 kcal AMEn/kg; 22% CP; 11.6% Lys.

R1: Reduction 1 diet: control diet – 60 kcal/kg; −2.3% aa.

R2: Reduction 2 diet: control diet – 120 kcal/kg; −4.6% aa.

W-B-S: Wheat, barley and soy based diet.

C-S: Corn and soy based diet.

a–b–c Means with different superscripts in a column differ significantly (*P* ≤ 0.05).

**Table 8:** Effect of nutrient density reduction and protected sodium butyrate (PSB) inclusion on different variables of intestinal morphology of growing chickens at d 21 (Trial 1, 2, and 3).

		Villi length, microns			Villus:Crypt ratio			Mucosa thickness, microns		
		Trial 1	Trial 2	Trial 3	Trial 1	Trial 2	Trial 3	Trial 1	Trial 2	Trial 3
		Ileum 21 d	Ileum 21 d	Ileum 21 d	Ileum 21 d	Ileum 21 d	Ileum 21 d	Ileum 21 d	Ileum 21 d	Ileum 21 d
Control	NO	392	453	396	2.8	3.0	4.2	536	603	525
R 1	NO	402	494	420	2.7	3.5	4.8	549	634	521
R 2	NO	-	-	445	-	-	4.7	-	-	567
C	PSB	394	486	444	2.8	3.0	4.7	533	646	596
R 1	PSB	376	541	443	2.3	3.6	5.0	538	692	573
R 2	PSB	-	-	472	-	-	5.4	-	-	599
SEM		35.9	21.5	20.6	0.25	0.16	0.28	40.1	23.6	24.3
*P*-value (Nutrient × Additive)		NS	NS	NS	NS	NS	NS	NS	NS	NS
Main effect mean										
Nutrient density	C	389	470	420	2.5	3.0	4.4^y^	544	624	561
	R 1	393	518	432	2.8	3.5	4.9^xy^	535	663	547
	R 2	-	-	458	-	-	5.0^x^	-	-	583
SEM		25.4	15.2	14.6	0.18	0.11	0.20	28.4	16.7	17.2
*P*-value (Nutrient density)		NS	0.039	0.169	NS	0.004	0.086	NS	0.118	NS
PSB addition	NO	397	474	420	2.7	3.2	4.5	543	618	538
	PSB	385	514	453	2.5	3.3	5.0	536	669	590
SEM		25.4	15.2	11.9	0.18	0.11	0.16	28.4	16.7	14.0
*P*-value (PSB addition)	Additive	NS	0.080	0.056	NS	NS	0.033	NS	0.047	0.009

PSB: Protected sodium butyrate at 1 kg/t of feed.

C: Control diet: 3,000 kcal AMEn/kg; 22% CP; 11.6% Lys.

R1: Reduction 1 diet: control diet – 60 kcal/kg; −2.3% aa.

R2: Reduction 2 diet: control diet – 120 kcal/kg; −4.6% aa.

W-B-S: Wheat, barley and soy based diet.

C-S: Corn and soy based diet.

x–y Means with different superscripts in a column tend to differ (*P* ≤ 0.10).

**Table 9:** Effect of nutrient density reduction and protected sodium butyrate (PSB) inclusion on *Lactobacilli* and *E. Coli* concentrations (log10 CFU/g) of intestinal content of growing chickens at 21 and 42 d.

		Trial 1 W-B-S				Trial 2 C-S			
		*Lactobacilli*	*Lactobacilli*	*E. Coli*	*E. Coli*	*Lactobacilli*	*Lactobacilli*	*E. Coli*	*E. Coli*
		Ileum 21 d	Ileum 42 d	Caecum 21 d	Caecum 42 d	Ileum 21 d	Ileum 42 d	Caecum 21 d	Caecum 42 d
Control	NO	7.54	7.81	7.60	9.09	8.25	7.98	8.59	7.76
R 1	NO	7.64	7.45	7.37	8.93	7.30	7.76	8.66	8.02
C	PSB	7.32	7.51	7.89	8.87	7.62	7.61	7.89	7.80
R 1	PSB	7.58	7.68	7.16	9.05	7.68	7.88	8.04	7.67
SEM		0.403	0.196	0.236	0.308	0.283	0.332	0.235	0.288
*P*-value (Nutrient × Additive)		NS	NS	NS	NS	NS	NS	NS	NS
Main effect mean									
Nutrient density	C	7.43	7.66	7.75	8.98	7.54	7.82	8.46	7.88
	R 1	7.61	7.57	7.26	8.99	8.04	7.84	8.37	7.79
SEM		0.285	0.139	0.167	0.218	0.199	0.235	0.166	0.204
*P*-value (Nutrient density)		NS	NS	0.057	NS	NS	NS	NS	NS
PSB addition	NO	7.59	7.63	7.48	9.01	8.00	7.88	8.63	7.91
	PSB	7.45	7.60	7.52	8.96	7.65	7.77	7.98	7.74
SEM		0.285	0.139	0.167	0.218	0.199	0.235	0.166	0.204
*P*-value (PSB addition)		NS	NS	NS	NS	NS	NS	0.007	NS

PSB: Protected sodium butyrate at 1 kg/t of feed.

C: Control diet: 3,000 kcal AMEn/kg; 22% CP; 11.6% Lys.

R1: Reduction 1 diet: control diet – 60 kcal/kg; −2.3% aa.

W-B-S: Wheat, barley and soy based diet.

C-S: Corn and soy based diet.

**Table 10:** Effect of nutrient density reduction and protected sodium butyrate (PSB) inclusion on volatile fatty acid concentrations of trial 1 (mM) of growing chickens at d 21.

Nutrient	Additive	Ileum				Caecum			
		Lactic acid	Acetic acid	Propionic acid	Butyric acid	Lactic acid	Acetic acid	Propionic acid	Butyric acid
C	NO	42.96	3.67^b^	1.80	0.002	0.92	90.78^B^	10.39^b^	13.80^ab^
R 1	NO	28.50	13.09^ab^	2.01	0.007	5.64	145.05^A^	16.08^ab^	21.19^a^
C	PSB	49.50	16.38^a^	2.65	0.003	1.55	166.66^AB^	17.50^a^	18.32^ab^
R 1	PSB	35.58	8.72^ab^	1.89	0.671	2.57	84.56^B^	12.47^ab^	12.97^b^
SEM		9.256	3.234	0.304	0.286	1.410	13.149	2.090	2.617
*P*-value (Nutrient × Additive)		NS	0.017	0.124	NS	NS	0.005	0.020	0.027
Main effect mean									
Nutrient density	C	46.23	10.02	2.23	0.002	1.24	103.72	13.95	16.07
	R 1	32.04	10.91	1.95	0.339	4.11	114.81	14.28	17.09
SEM		6.545	2.287	0.215	0.203	0.998	9.305	1.478	1.851
*P*-value (Nutrient density)		0.144	NS	NS	NS	0.060	NS	NS	NS
PSB addition	NO	35.73	8.38	1.91	0.004	3.28	117.92	13.24	17.50
	PSB	42.54	12.55	2.27	0.337	2.06	100.61	14.99	15.65
SEM		6.545	2.287	0.215	0.203	0.998	9.305	1.478	1.851
*P*-value (PSB addition)		NS	NS	NS	NS	NS	NS	NS	NS

PSB: Protected sodium butyrate at 1 kg/t of feed.

C: Control diet: 3,000 kcal AMEn/kg; 22% CP; 11.6% Lys.

R1: Reduction 1 diet: control diet – 60 kcal/kg; −2.3% aa.

W-B-S: Wheat, barley and soy based diet.

C-S: Corn and soy based diet.

a–b Means with different superscripts in a column differ significantly for that main effect (*P* ≤ 0.05).

A–B Means with different superscripts in a column differ significantly for that main effect (*P* ≤ 0.01).

**Table 11:** Effect of nutrient density reduction and protected sodium butyrate (PSB) inclusion on crude protein retention (%), retained protein (g/kg), gross energy metabolizability (%) and retained energy (kcal/kg) of growing chickens of 21 d of age (Trial 3: wheat, barley, soy based diets).

		Protein retention (%)	Retained protein (g/kg)	Energy (%)		Retained energy (kcal/kg)
Control	NO	68.57	151.00	70.79		2,124
R 1	NO	67.34	148.12	69.59		2,046
R 2	NO	65.02	142.66	69.43		2,000
C	PSB	64.80	142.68	72.98		2,189
R 1	PSB	71.19	156.48	73.51		2,161
R 2	PSB	66.59	146.10	71.17		2,050
SEM		1.758	3.866	1.322		39.0
*P*-value (Nutrient × Additive)		0.103	0.102	NS		NS
Main effect mean						
Nutrient density	C	66.68	146.84	71.89		2,156^A^
	R 1	69.29	152.30	71.55		2,103^AB^
	R 2	65.81	144.38	70.30		2,024^B^
SEM		1.243	2.733	0.935		27.6
*P*-value (Nutrient density)		NS	NS	NS		0.0074
PSB addition	NO	66.99	147.26	69.94		2,056
	PSB	67.53	148.42	72.55		2,133
SEM		1.015	2.232	0.763		22.5
*P*-value (PSB addition)		NS	NS	0.0216		0.0219

PSB: Protected sodium butyrate at 1 kg/t of feed.

C: Control diet: 3,000 kcal AMEn/kg; 22% CP; 11.6% Lys.

R1: reduction 1 diet: control diet 60 kcal/kg; −2.3% aa.^c^

c ^A-B^ Means with different superscripts in a column differ significantly for that main effect (*P* ≤ 0.01).

#### Trials 1 and 2

Two hundred 1-day-old male Cobb broiler chickens were divided into 20 pens with 10 animals per pen (5 replicates per treatment), in a completely randomized design. The trials lasted 42 d. The nutritional program consisted of 2 phases: starter (0–1 d) and grower (21–42 d) feeds. The dietary treatments were arranged in a 2 × 2 factorial design (4 treatments) with 2 dietary formulations (C and R1) with or without the inclusion of 1 kg/t of PSB. Birds were weighed at d 21 and 42. Feed intake per pen was measured weekly throughout the study.

#### Trial 3

Two hundred and fifty-two 1-day-old male Cobb broiler chickens were divided into 36 pens with 7 animals per pen (6 replicates per treatment), following a completely randomly assigned distribution. The nutritional program consisted of 2 phases: starter (0–21 d) and grower (21–42 d). The dietary treatments were arranged in a 3 × 2 factorial design (6 treatments) with 3 dietary formulations (C, R1 and R2) with or without the inclusion of 1 kg/t of PSB. Birds were weighed weekly and individually identified using numbered wing bands at d 7. Feed supplied per pen was measured weekly throughout the study. At d 14, three birds per pen were transferred to digestibility crates (36 crates in the overall study, one per pen). Selection of birds was done based on their weights in order to have a representative sample from every pen. After being weighed at d 14, birds within every pen were paired by the closest weight. In pens with 7 birds, the bird that its difference of weight was greater (higher or lower) than the others was unpaired and excluded from the selection process. A bird from each pair was selected at random. Leftover birds remained in their pens until the end of the study (study d 42). The digestibility crate phase lasted 7 d, from d 14 to 21. First 4 d (14–18) was an acclimatization period. Total excreta was collected afterward and weighed daily on d 19, 20, and 21. Feed intake was measured per crate during the acclimatization and collection period (d 19–21). At d 22, all birds in digestibility crates were individually weighed and euthanized to sample duodenum and ileal sections for gut morphological study.

#### Trial 4

A total of 2,208 one-day-old male Ross 708 broiler chicks were included in this trial. Broilers were allocated to 48 pens, with 46 birds/pen and 8 replicates/treatment, in a completely randomized design. The nutritional program consisted of 3 diets: starter (0–14 d), grower (14–28 d) and finisher (28–42 d) fed from 1 to 42 d of age. The study was conducted on recycled litter from a prior broiler experiment, as the use of recycled litter is quite a common practice in North America. The dietary treatments were arranged in a 3 × 2 factorial design (6 treatments) with 3 dietary formulations (C, R1 and R2) with or without the inclusion of 0.1% of PSB.

### Analyses

#### Microbiota Determinations

In trials 1 and 2, at d 21 and 42, one animal per pen was euthanized and samples of duodenum, jejunum, ileum, and caecum were extracted to analyze *Lactobacilli*, coliforms and *E. coli* populations by plating. *Lactobacilli*, coliforms and *E. coli* in the intestinal contents were analyzed diluting 3 g of sample into 300 mL of sterile saline solution (0.9%) + Tween 80 (0.4%) and homogenized in a sterile mincer. The diluted sample was homogenized at 11,200 G-force for 1 min. A series of dilutions were prepared by pipetting 1 mL of the latest diluted sample into 9 mL of the saline solution. Colony forming units (**CFU**) were counted after culturing in Petri plates with the corresponding culture media: MRS Agar for *Lactobacilli*, MacConkey Agar for coliforms and TBX for *E. coli*. The plates were incubated for 48 h at 37°C. Only plates containing between 30 and 300 cfu were considered. Results were obtained from at least 3 independent measurements.

#### VFA Analysis

In trials 1 and 2, at d 21 and 42, one animal per pen was euthanized and samples of duodenum, jejunum, ileum, and caecum were extracted to analyze volatile fatty acids concentrations, VFA of chicken digesta were analyzed dissolving and homogenizing 1 g of sample with 2 mL of buffer pH 2.0 in a test tube. After 10 min at 2,800 G-force centrifugation, an aliquot of the supernatant was filtered through a 0.45-mm pore nylon membrane and injected into the HPLC (HPLC Agilent 1200 series, VWD/DIR detector (Agilent Technologies, Inc., Santa Clara, CA), with data acquisition system: Agilent ChemStation (+software) and column Zorbax SB-Aq, 4.6 mm × 150 mm × 5 µm). Two chromatographic profiles were obtained, one corresponding to the UV-VWD signal, and another profile corresponding to the DIR signal. This second profile (DIR) was used to quantify the butyric acid, and the first profile (UV-VWD) to quantify the remaining acids. This is because in the UV-VWD profile, at the retention time of the butyric acid, there are also many components that absorb at 210 nm, and this causes errors in the quantification of this acid.

#### Intestinal Morphology Determinations

In trials 1 and 2, at d 21 and 42, one animal per pen was euthanized and samples of duodenum, jejunum, ileum, and caecum were extracted to analyze intestinal villi morphology. In trial 3, the 3 animals/replicate that had been selected for the digestibility evaluation were euthanized for the intestinal morphology evaluation. Birds were euthanized by cervical dislocation. Birds were euthanized between 2 and 3 h after switching on the lights to ensure that digestive tract was filled. All samples were identified with an individual code and the study code. Samples taken were approximately 3 cm of duodenum and jejunum sections and 5 cm of ileum section (at 5–10 cm of the ileocecal junction). To remove residual contents for a better microscopy observation and ensuring a correct penetration of 10% neutral-buffered formaldehyde solution (**NBF**), a longitudinal section of about 0.5 cm was made on each extreme and samples were gently flushed with 10% NBF. All samples were stored into sterile plastic containers with 10% NBF at ratio 1:10. The crypt and villi length were determined with micrographs taken to 3 microns histological sections, obtained from transversal cuts of every intestine section, and stained with Hematoxiline-Eosine. Micrographs were enlarged ×100 (Leica DM1000 microscope, Leica ICC50 HD camera; Leica, Wetzlar, Germany). In those pictures, the 10 longest villi were measured, as well as 10 crypts that could be observed completely. Mucosa thickness is the result of adding villi length and crypt depth. To evaluate villi and crypt density, number of villi and crypts are counted in each distance (between 1,200 and 2,500 microns; in this case, all crypts (complete or incomplete) were included in the sum. The number is divided by the length, obtaining villi/mm or crypt/mm.

#### Crude Protein Retention and Energy Metabolizability Determinations

Total excreta was daily collected, weighed, frozen, and oven dried at 103°C until constant weight per digestibility crate on d 19, 20, and 21. Before chemical analysis, the fecal samples were thawed and finely ground to a size that could pass through a 1-mm screen. All feed samples were collected at the beginning of the trial, and the average value of analyzed composition was used to represent feed composition and calculations.

All feed and fecal samples were analyzed for dry matter (**DM**), Ashes, gross energy (**GE**), and crude protein (**CP**) following the procedures outlined by the Association of Official Analytical Chemists (International, 1995). The DM was determined on an aliquot sample to establish the residual water content after drying for 24 h at 100°C and the ash content was determined after ignition of a weighed sample in a muffle furnace (Carbolite CWF 1100; Fisher Scientific, Waltham, MA) at 550°C for 6 h. The corresponding analytical result was expressed on a DM basis. The GE content of diets and fecal samples was determined using an adiabatic bomb calorimeter (model 356, Parr Instrument Company, Moline, IL). The CP of diets and feces were determined by the Kjeldhal system (Kjeltec 8400 Analyzer Unit, FOSS, Hillerød, Denmark). The CP was determined as total N × 6.25.

Crude protein retention (**CPr**) and gross energy metabolizability (**GEm**) were determined by the difference between nutrients in the feed and nutrients in the feces; GE of feces was corrected by nitrogen content.

#### Statistical Analysis

All data were analyzed as a 2-way ANOVA using the GLM procedure of the SAS system (9.4, SAS Institute Inc., Cary, NC). The model included the main effect of diets, sodium butyrate, and their interaction. Pen was considered as an experimental unit. The means showing significant (*P* ≤ 0.05) treatment differences in the ANOVA were then compared using Tukey's test. All data were tested for normality and homogeneity of variances, using the UNIVARIATE procedure and Bartlett test of SAS (9.4), respectively.

## RESULTS

### Effects on Performance

Table 6 shows animal performance results from 1 to 21 or 28 d of age. Nutrient density reduction had influenced body weight (**BW**) in trial 3 (3% lower weight for R2, *P* = 0.03) and in trial 4 (2.2 and 5.8%, reduction of BW for R1 and R2, respectively *P* < 0.001) and it impaired FCR in trial 3 (1% and 4%, *P* = 0.003) and in trial 2 (3%, *P* = 0.0972). The addition of PSB made the animals gain more in all 4 trials (*P* < 0.05), and improved FCR in both trials with WBS based diets (trial 3, 3.2%, *P* 0.003 and trial 1, 2.6%, *P* = 0.0912).

The interaction between nutrient concentration and additive was only significant in trial 4 for the BW of the animals. PSB animals’ weight did not decrease with the reduction of nutrient concentration of diets as it did when the animals did not receive the additive (*P* = 0.004).

Cumulative performance results are detailed in Table 7. Nutrient concentration affected final BW only in trial 4 (2.5 and 5.7% decreased *P* < 0.001, respectively as the nutrient concentration was reduced 2.3 and 4.6%, respectively); it also impaired FCR in trials 2 (2.5%, *P* = 0.03) and 4 (1.8 and 5.6%, *P* < 0.001), both of which were CS feed-based trials. The addition of sodium butyrate made the animals grow more in trials 2 (7.3%, *P* = 0.004) and 4 (1.9%, *P* = 0.003). In trials 1 and 4, there was an interaction between nutrient density and protected sodium butyrate addition. In trial 1, PSB made the animals grow more when added to R1 diet (6.5%) and there was no difference when added to the control diet. In trial 4, final BW decreased as nutrient concentrations reduced, whereas when R1 was supplemented with PSB, final BW of chicken was not significantly different to control.

### Effects on Intestinal Morphology

The effect of treatments on intestinal morphology was assessed in duodenum, jejunum, and ileum by means of villi and crypt length measures; however, only ileum data determined in chickens of 21 d of age are shown in Table 8. Data of trial 4 are not shown because they were measured at different age and no effect of treatments was observed. Few significant effects of treatments on intestinal morphology were observed. The decrease in nutrient density produced 41 microns longer villi at d 21 in the ileum in trial 2 (*P* = 0.039) and higher villus:crypt ratio (3 vs. 3.5; *P* = 0.0045). The same happened in trial 3 (0.5 and 0.6 higher villus:crypt ratio in R1 and R2 respect to control diet, respectively, *P* = 0.086). In the same way, chickens that received the R1 diet had 39 microns thicker mucosa in the ileum in trial 2 (*P* = 0.1184). The inclusion of PSB showed an increase of villi length in trials 2 (40 microns longer, *P* = 0.08) and 3 (33 microns longer; *P* = 0.05); also, it improved villus:crypt ratio in trial 3 (0.5 higher, *P* = 0.0337), and produced a thicker mucosa in trials 2 (51 microns, *P* = 0.0479) and 3 (42 microns, *P* = 0.009).

There was an interaction between reduction of nutrient concentration and the addition of PSB in the jejunum morphology at d 42 in trial 1. There were no differences between C and R1 when the diets were not supplemented with PSB, however, PSB produced longer villi length, higher villus:crypt ratio and thicker mucosa in R1 than in C (data not shown, *P* = 0.02).

### Effects on Intestinal Microbiology

There were scarce effects of treatments on the intestinal microbiology evaluated. The reduction of nutrient concentration tended to decrease *E. coli* population in the caecum (6.3%, *P* = 0.0572) at d 21 in trial 1, and to increase *Lactobacilli* counts in the ileum in trial 2 (6.6%; *P* = 0.11). The addition of PSB decreased *E. coli* concentration by 7.6% in the caecum at d 21 in trial 2 (*P* = 0.007). There was no significant interaction between nutrient density and the addition of PSB, as can be seen in Table 9.

### Effects on Volatile Fatty Acids

Table 10 contains results of volatile fatty acid concentration of trial 1. Results of trial 2 were inconclusive. The nutrient concentration reduction of the diet tended to increase lactic acid concentration in the caecum (*P* = 0.060). The addition of PSB did not have any effect on any of the VFA evaluated. However, the interaction of nutrient concentration and PSB addition was significant in ileum for acetic (*P* 0.017), where PSB produced higher concentration in chickens fed the C diet than the R1. This interaction was also observed in the caecum, where acetic acid (*P* 0.005), propionic acid (*P* 0.020), and butyric acid (*P* 0.027) increased with the reduction of nutrient concentration of nonsupplemented diets and decreased in PSB supplemented ones.

### Effects on Energy and Protein Retention

In trial 3, chickens that received diets supplemented with PSB showed 2.66% higher (*P* = 0.021) ME (Table 11). Crude protein retention tended to be impaired with the reduction of nutrient concentration when the animals were not supplemented with PSB, but that effect tended to disappear with the addition of PSB (*P* = 0.102).

## DISCUSSION AND CONCLUSIONS

Both factors evaluated in the trials, energy and amino acid concentration and presence or absence of PSB, had effects on broilers performance and GIT parameters. The interaction between these factors was significant in several parameters.

On one hand, the reduction of energy and amino acid concentration increased 4 to 6% bird FCR, as described by Houshmand et al., 2011. Houshmand et al., 2011 explained how a reduction in the energy concentration produced a lower protein retention in the animals. Besides, the reduction of key nutrients also affects the growth (Angel et al., 2005; Zhao and Kim, 2017), as animals do not receive enough nutrients to achieve potential growth.

In these trials the reduction of energy and amino acid concentration had a positive effect on the GIT development (longer villi, higher villi:crypt ratio [**V:C**], and thicker mucosa). Yamauchi et al. (1993) reported that a reduction in the energy density of the diet produced longer villi and ameliorated gastrointestinal epithelium development, because the animals needed more intestinal surface to compensate for the lack of nutrient concentration. Laudadio et al. (2012), observed how reducing 2% dietary protein concentration produced longer villi in broilers, as well as a higher V:C ratio. These authors described how a higher V:C is related to a slower turnover of the intestinal epithelium and therefore with lower maintenance requirements. Besides, Boontiam et al. (2017) explained that a higher level of mitosis can produce longer villi and a lower V:C ratio when energy and protein are reduced in the diet. Just the same effects that Houshmand et al., 2011 published; besides, Zou et al. (2013) and Miao et al. (2017) reported shorter ileal villi with lower energy concentration, but higher V:C ratio, and related these results to an improvement of the mechanism of absorption of different nutrients. These results are the opposite to Fosoul et al. (2018) reported, who did not find any difference in intestinal morphology with diets differing in nutrient concentration or Chen et al. (2019), who saw shorter villi in jejunum.

It seems that when diets do not supply the adequate nutrient density, the animal will try to compensate by improving the gastrointestinal epithelium development (wider surface of absorption) and the efficiency of nutrient absorption.

The addition of PSB to poultry diets has a positive effect on the performance of broilers as obtained in this work (Guilloteau et al., 2010). On one hand, PSB addition improved the FCR by 3.7%, (trials 1 and 3) similarly to results obtained by Chamba et al. (2014) and Sikandar et al. (2017). On the other hand, PSB produced 2.8% heavier birds, as reported by Lan et al. (2020). Guilloteau et al. (2010) described very well the main reasons why sodium butyrate improves animal performance; it has effects on the gastrointestinal tract development and ambience (microbiota, volatile fatty acids, etc.), on feed digestibility, and even effects on animals health, reducing the incidence of subclinical illnesses ( Jerzsele et al., 2012), altering immune response (Bortoluzzi et al., 2017); and helping the animals cope better with infectious challenges (Fernández-Rubio et al., 2009; Liu et al., 2019; Bortoluzzi et al., 2018).

In this set of trials, the addition of protected sodium butyrate showed many of the effects described in the literature: It improved GIT epithelium development in the small intestine (Guilloteau et al., 2010; Jerzsele et al., 2012; Liu et al., 2019), as it is used very efficiently by the gastrointestinal tract cells as a source of energy, it produced longer villi, higher villus:crypt ratio (related to a more efficient intestinal turnover) and thicker mucosa. Besides, butyrate had an effect on microbial populations along the gastrointestinal tract, steering its predictive function; Bortoluzzi et al. (2017) reported how microbial populations changed when energy and amino acids were reduced in the diet, because it needed to adapt to the nutrients available, when butyrate was added to the diet, many of the changes in the populations, related to fiber fermentation (and hence volatile fatty acid production) did not happen, keeping its function as in the control diets. Zhu et al. (2015) described how changes in bacterial populations vary the volatile fatty acid concentrations in the caecum of broilers, as the different bacteria species follow different metabolic pathways to produce energy, just as was seen in trial 1, where the volatile fatty acid production was similar between the control and the reduced diet supplemented with sodium butyrate. Lastly, PSB improved energy digestibility, because of the bigger surface of absorption related to the better gastrointestinal epithelium development and by activating protein receptors, that increase the expression of transporters within enterocytes, ultimately affecting FCR (Mallo et al., 2012; Bortoluzzi et al., 2017; Liu et al., 2019).

When the interaction between nutrient density and the addition of PSB was evaluated, it is interesting to see how when PSB is added to the nutrient reduced diets at 1 kg/t, it helped the animals to achieve a final body weight similar to the control diets, with higher nutrient concentration, without the additive. In this set of trials, the interaction showed differences in volatile fatty acid concentrations in the gastrointestinal tract, these differences are probably due to changes in bacterial populations different from the ones analyzed in the trials, as described by Bortoluzzi et al. (2017). Also, studying the interaction between nutrient density and PSB for Protein Retention %, it was observed that PSB tended to avoid the linear Protein Retention % impairment related to the reduction of nutrient density, as this parameter behaved differently in the PSB treatments. PSB helped the animals to better utilize the nutrients available in the diet through a better intestinal morphology and a more stable intestinal microbiota.

It can be concluded that the use of PSB helps the animals cope better with adverse situations like nutrient dilution, and it does it independently of the ingredients used in the diet. This may open the door to change the main ingredients in the diets and to the use of nonconventional raw materials, local, less evaluated and with uncertain quality, as the animal will be better prepared to use the nutrients available in the feed.

## DISCLOSURES

The authors have no conflicts of interest to report.
